# Emergence of Small-World Anatomical Networks in Self-Organizing Clustered Neuronal Cultures

**DOI:** 10.1371/journal.pone.0085828

**Published:** 2014-01-28

**Authors:** Daniel de Santos-Sierra, Irene Sendiña-Nadal, Inmaculada Leyva, Juan A. Almendral, Sarit Anava, Amir Ayali, David Papo, Stefano Boccaletti

**Affiliations:** 1 Center for Biomedical Technology, Universidad Politécnica de Madrid, Pozuelo de Alarcón, Madrid, Spain; 2 Complex Systems Group, Universidad Rey Juan Carlos, Móstoles, Madrid, Spain; 3 Department of Zoology, Tel-Aviv University, Tel Aviv, Israel; 4 Istituto dei Sistemi Complessi, Consiglio Nazionale delle Ricerche, Sesto Fiorentino, Florence, Italy; 5 Istituto Nazionale di Fisica Nucleare, Sesto Fiorentino, Florence, Italy; University of Maribor, Slovenia

## Abstract

*In vitro* primary cultures of dissociated invertebrate neurons from locust ganglia are used to experimentally investigate the morphological evolution of assemblies of living neurons, as they self-organize from collections of separated cells into elaborated, clustered, networks. At all the different stages of the culture's development, identification of neurons' and neurites' location by means of a dedicated software allows to ultimately extract an adjacency matrix from each image of the culture. In turn, a systematic statistical analysis of a group of topological observables grants us the possibility of quantifying and tracking the progression of the main network's characteristics during the self-organization process of the culture. Our results point to the existence of a particular state corresponding to a *small-world* network configuration, in which several relevant graph's micro- and meso-scale properties emerge. Finally, we identify the main physical processes ruling the culture's morphological transformations, and embed them into a simplified growth model qualitatively reproducing the overall set of experimental observations.

## Introduction

The issue of why and how an assembly of isolated (cultured) neurons self-organizes to form a complex neural network is a fundamental problem [1]–[3]. Despite their more limited, and yet laboratory-controllable, repertoire of responses [1], [4], the understanding of such cultures' organization is, indeed, a basis for the comprehension of the mechanisms involved in their *in vivo* counterparts, and provide a useful framework for the investigation of neuronal network development in real biological systems [3].

Some previous studies highlighted the fact that the structuring of a neuronal cultured network before the attainment of its mature state is not random, being instead governed and characterized by processes eventually leading to configurations which are comparable to many other real complex networks [5]. In particular, networking neurons simultaneously feature a high overall clustering and a relatively short path-length between any pair of them [6]. Such configurations, which in graph theory are termed *small-world* [7], are ubiquitously found in real-world networking systems. Small-world structures have been shown to enhance the system's overall efficiency [8], [9], while concurrently warranting a good balance between two apparently antagonistic tendencies for segregation and integration in structuring processes, needed for the network's parallel, and yet synthetic performance [10].

In this paper, we experimentally investigate the self-organization into a network of an *in vitro* culture of neurons during the course of development, and explore the changes of the main topological features characterizing the anatomical connectivity between neurons during the associated network's growth. To that purpose, dissociated and randomly seeded neurons are initially prepared, and the spontaneous and self-organized formation of connections is tracked up to their assembling into a two dimensional clustered network.

Most existing studies in neuronal cultures restricted their attention to functional networks (statistical dependence between nodes activities) and not to the physical connections supporting the functionality of the network [11]. The reason behind this drawback is that the majority of investigations focused on excessively dense cultures, hindering the observation of their fine scale structural connectivity. Although there are studies striving to indirectly infer the underlying anatomical connectivity from the functional network, it has been shown that strong functional correlations may exist with no direct physical connection [12]. Only few studies dealt with the physical wiring circuitry. However, on the one hand, only small networks were considered; on the other hand, how the network state evolves during the course of the maturation process has not been investigated [6].

Here, instead, we focus on intermediate neurons' densities, and provide a full tracking of the most relevant topological features emerging during the culture's evolution. In particular, we show experimentally that *in vitro* neuronal networks tend to develop from a random network state toward a particular networking state, corresponding to a *small-world* configuration, in which several relevant graph's micro- and meso-scale properties emerge. Our approach also unveils the main physical processes underlying the culture's morphological transformation, and allows using such information for devising a proper growth model, qualitatively reproducing the set of our experimental evidence.

Together with confirming several results of previous works on functional connectivity [13], or on morphological structuring at a specific stage of the cultures' evolution [6], we offer a systematic characterization of several topological network's measures from the very initial until the final state of the culture. Such a *longitudinal* study of the network structure highlights as yet unknown self-organization properties of cultured neural networks, such as *i*) a large increase in both local and global network's efficiency associated to the emergence of the small-world configuration, and *ii*) the setting of assortative degree-degree correlation features.

## Experimental Set-Up

### Neuronal cultures and network growth

In this paper, we report on six cultured networks, which were grown from independent initial sets of dissociated neurons extracted from the frontal ganglion of adult locusts of the *Schistocerca gregaria* species. In all cases, a same protocol was used, involving animals that were daily fed with organic wheat grass and maintained under a 12∶12 h light∶dark cycle from their fifth nymph growth to their early adult stage of development. At this latter stage, we followed the dissection and culturing protocol thoroughly described in [14]. In brief, the frontal ganglia were dissected from anesthetized animals, and enzymatically treated to soften the sheath. Ganglia were then forced to pass through the tip of a 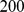 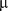
l pipette to mechanically dissociate the neurons. The resulting suspension of neuronal somata was plated on Concanavalin A pre-coated circular area (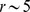 mm) of a Petri dish where it was left for 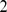 h to allow adhesion of neurons at random positions of the substrate. After plating, 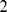 ml culture medium (Leibovitz L-15) enriched with 5% locust hemolymph was added. Cultures were then maintained in darkness under controlled temperature (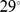
C) and humidity (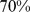).

The density at which cultures are seeded determines the maturation rate and the spatial organization at the mature state [15], [16]. For the purpose of this work, aimed at studying the network evolution into a clustered network, 6 dense cultures of 12 ganglia each (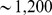 neurons) were used and monitored during 18 days *in vitro* (DIV). During the entire experiment, the culture medium was not changed.

High-resolution and large scale images of the whole culture were acquired daily using a charge coupled device camera (DS-Fi1, Nikon) mounted on a phase contrast microscope (Eclipse Ti-S, Nikon), with automated control of a motorized XYZ stage (H117 ProScan, Prior Scientific).

**Figure 1:**
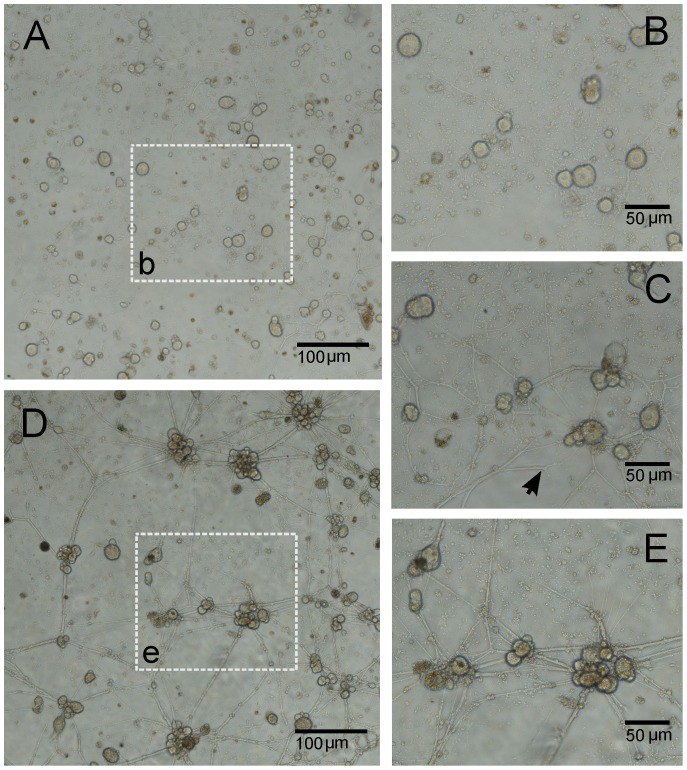
Culture development of locust frontal ganglion neurons into clustered networks. (A) After 3 DIV, completely dissociated neurons had already started growing neuronal processes with continuous branching. The area outlined in (b) is enlarged in B. (C) Same area as in (B) but at 6 DIV. At this stage, neurons and small clusters of neurons are already densely connected and form a complex network. At the same stage, branched neurites (pointed by the black arrow) that failed to contact neighboring neurons start to retract. (D) Migration of neurons due to the tension along neurites leads to the formation of large neuronal clusters and of thicker bundles of neurites. For a better visualization, the area outlined in (e) is enlarged in E.

A typically observed growth evolution is shown in Fig. 1 (restricted to just a small part of the whole culture) between 3 and 12 DIV. Neurons ranging from 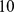 to 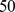 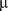
m in size are initially randomly anchored to a two dimensional substrate, while after 3 DIV (Fig. 1A and B) many cells already start growing neuronal processes (neurites) trying to target neighboring cells. During this growth process, neurites also split and reach other processes forming loops of neurites up to 6 DIV, when the maximum stage of network development takes place (Fig. 1C). At this point, the growth rate decreases and a different mechanism starts shaping the network: tension is generated along the neurites as they stretch between neurons or bifurcation points to form straight segments [17].

The latter process favors neuron migration, giving rise to clusters of neurons, and the fusion of parallel neurites into thicker bundles together with the retraction of those branches which did not target any neuron (see black arrow in Fig. 1C). The resulting network topology shown in Fig. 1D after 12 DIV (and in the enlarged area in Fig. 1E) is characterized by a random distribution of few clusters of aggregated neurons linked by thick nerve-like bundles.

### Anatomical graph extraction and complex network statistics

Our experiments consistently show that cultures self-organize from random scattered distributions of bare neurons into spatial networks of interconnected clusters of neurons (compare Fig. 1A and Fig. 1E).

**Figure 2:**
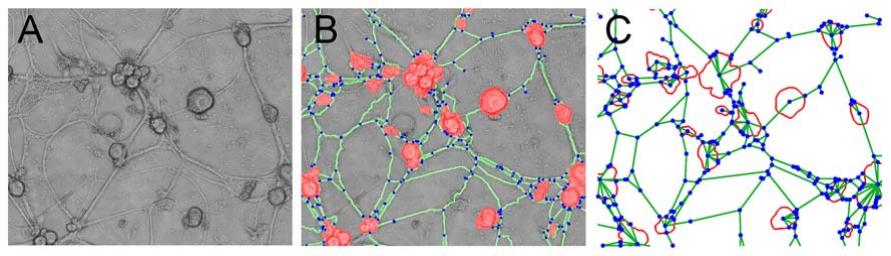
Extraction of the adjacency matrix defining the neural network connectivity. (A) Image cut taken from a 6 DIV culture and (B) the layer on top showing the identification of neurons and clusters of neurons (red), neurites connecting them (green) and neurite branching points (blue). (C) Mapping of the neuronal network into a graph where blue dots represent the nodes and green lines the links of the graph.

In order to properly quantify the topological and spatial changes of the anatomical neuronal network as cultures approach their mature state, we developed a custom image analysis software in MATLAB to detect the location of neurons, clusters of neurons and neurite paths. The used imaging software has been fully customized for the purpose of the analysis of the present data. The general details of the developed imaging software will be reported elsewhere. The performance of the algorithm is sketched in Fig. 2. The algorithm takes as an input a gray color image of the culture at a particular day (Fig. 2A), upon which it superimposes a layer of new information comprising the contours of the clusters of neurons (red shadows), the traces of the neurites (green lines), and connection points between neurites, as well as those between neurites and clusters (blue dots) (Fig. 2B).

The information contained in the produced layer is then used to map the neuronal network into a graph 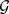 (see Fig. 2C) whose nodes (in blue) are either cluster centroids or connection points, and the links (in green) are straight lines connecting them. Therefore, our graph is made of two types of nodes: neurons or clusters of neurons (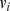) and neurite connection points (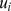). Treating all links as identical, i.e. ignoring edge length and edge directionality, this graph can be described in terms of a symmetric adjacency matrix 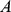 whose elements 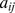 are equal to 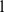 if nodes 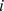 and 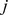 are linked, and 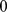 otherwise.

We focus on the network statistical properties at the level of the 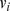 nodes, ignoring the dynamics of both neurite connections and branching points. Therefore, we extract from 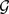 the subgraph defining the connectivity among nodes of class 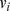 in such a way that 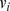 and 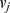 are linked either directly or through a connected path of 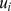 nodes.

**Figure 3:**
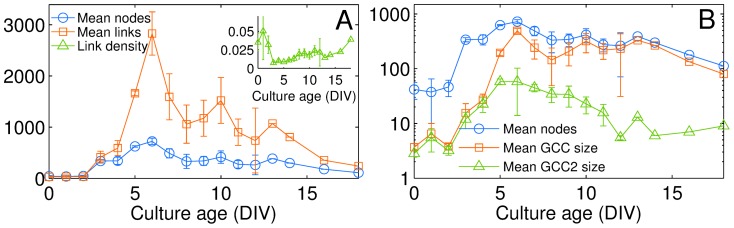
Density of the network as a function of culture age. (A) Mean number of nodes (blue circles), including neurons and clusters of neurons, and links connecting them (red squares), calculated for the 6 cultures vs. age (DIV). Inset: the link density (green triangles) quantifies the actual number of links divided by that of an all-to-all configuration [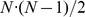, being 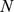 the number of connected nodes at each age]. (B) Log-linear plot of the mean number of nodes having at least one connection (blue circles), of the mean size of the giant connected component (red squares) and of the second largest connected component (green triangles). In all plots, error bars stand for the standard errors of the mean (sem).

The analysis of the networks' evolution requires accounting for the birth and death of links (and, in some cases, nodes) over time. Figure 3 shows the mean values for the number of nodes and the of links at each DIV, calculated for the 6 cultures. During the growth phase, spanning from 0 to 6 DIV, the number of nodes with at least one connection slowly increases with age, while the number of links rises exponentially, reaching a maximum at DIV 6. After this time point, the convergence of parallel neurites and neuronal clusterization induces a more gentle decrease in the number of links, accompanied by a slight reduction in the number of nodes. In order to properly compare networks of different size, we need to refer to a measure which is independent of the network size: the link density, defined as the ratio between the total number of measured links and the number of links characterizing the arrangement of the same number of identified nodes in a complete clique configuration. As illustrated in the inset of Fig. 3A, at any stage of development, the cultured networks are far from being fully connected (only about 2% of all possible connections exist between nodes), and thus operate in a low-cost regime of sparse anatomical connections.

In such a sparse connectivity regime, we quantify how our networks constrained in 2D space percolate. To do so, we measure the size 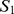 of the giant connected component (GCC) and the size 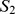 of the second largest component (GCC2) as a function of the age [18], [19]. Figure 3B shows that the number of nodes forming such connected components smoothly increases at the same rate along the first days of the network development, up to the DIV 6 when the difference in size between them suddenly and consistently starts to grow. From that point on, the GCC2 starts collapsing and progressively merging into the GCC, and the establishment of an almost fully connected network of clusters characterizes the rest of the culture's life. Figure 3B reports the evolution of the number of nodes belonging to both GCC and GCC2.

A deeper information on the culture evolution can be gathered by monitoring the behavior of a subset of local and network-wide quantities. For that purpose, we calculated several topological properties of the extracted adjacency matrices (using the Matlab Boost Graph Library package and the Brain Connectivity Toolbox [20]), whose definitions are provided in [5], [20]. In particular, we analyzed the clustering coefficient (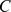), the average shortest path length (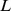), the local (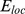) and global (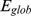) efficiency [8], the network assortativity (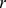) and the cumulative degree distribution (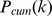), obtained from the degree distribution 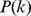 as 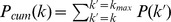 being 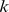 the degree (or number of links) of a node.

In all cases, the calculation of such statistics was restricted to the set of nodes having at least one link, and for the calculation of 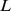 to those pairs of nodes belonging to the GCC. Moreover, the experimental values of 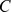 and 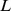 were also compared with those expected in equivalent random null hypothesis networks, i.e. random networks artificially constructed to have the same number of nodes and links and to display the same degree distribution. Specifically, for each experimental network at a particular age, we generated 20 independent realizations of equivalent random networks, and calculated the corresponding expected network statistics.

Finally, in order to quantify the degree-degree correlation properties, the network assortativity was defined by considering for each node 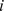 the average degree of its neighbors 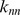, and by computing the linear regression of 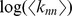 vs. 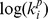. The assortativity coefficient 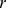 was then calculated as the Pearson correlation coefficient corresponding to the best fit of 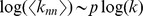. If 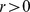 (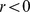), the network is set to be assortative (disassortative), while depending upon the obtained value of 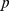, the degree correlation properties are said to be of a linear (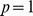), sub-linear (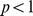), or super-linear (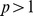) nature.

## Results

### Emergence of small-world structure

The first days of the cultures' development (from DIV 0 to DIV 3) were characterized by networks with very few nodes and links (see Fig. 3A). After DIV 3, the networks showed a very pronounced increase in the number of links and nodes (from DIV 3 to DIV 6) preceding a spatial network reorganization eventually driving the graph into its clustered, mature state.

**Figure 4:**
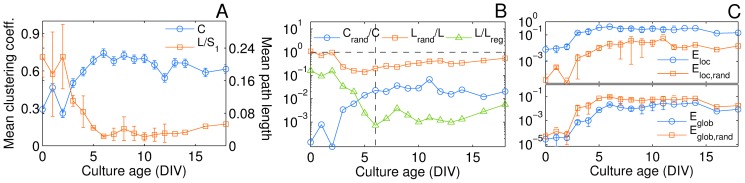
Network clustering and shortest path properties as a function of culture age. (A) Absolute values of the clustering coefficient 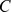 (blue circles, left axis) and mean path length 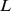 (red squares, right axis) normalized to the size of the largest cluster. (B) Semi-log plot of normalized values of 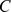 and 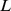 with respect to the expected values for an equivalent random network having the same number of nodes and links and preserving the degree distribution: 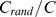 (blue circles) and 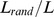 (red squares). The average path length is also compared to the value for a regular lattice as 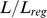 (green triangles) with 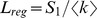, being 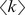 the average connectivity and 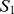 the size of the largest connected component. (C) Local (upper plot) and global (lower plot) efficiency as a function of culture age and compared to their respective values for the random graphs of the null model (see text for an explanation). All quantities are averaged for the set of 6 cultures at each day of measure (DIV). As in the Caption of Fig. 3, error bars represent the standard errors of the mean (sem).

The associated networks statistics sheds light on the transition from random to non-random properties with a progression of both the clustering coefficient and the average path length (normalized by the GCC size) as a function of age (see Fig. 4A). The first significant result is the simultaneous increase in the clustering coefficient and decrease in the mean path length, a clear fingerprint of the emergence of a small-world network configuration. This configuration becomes prominent at DIV 6 and stays relatively stable through the last two weeks *in vitro*. To properly asses the significance of this finding and isolate the influence of the variable network size and density, we calculated the values of 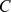 and 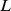 normalized to the corresponding expected values for equivalent random (and lattice) null model networks (see Fig. 4B). In doing so, we follow the approach that was recently used in similar circumstances for the obtainment of null models [21]. According to Watts and Strogatz's model [7], a small-world network simultaneously exhibits short characteristic path length, like random graphs, and high clustering, like regular lattices. Here, we found a clear change in the trend at DIV 6 where 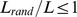, where the average path length of the cultured network starts to be close to that of a random graph and much smaller than that of a regular graph (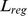 is calculated as 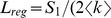)). At the same time, the clustering coefficient was much higher (between 30–50 times) than that of the corresponding random graphs.

These results are in agreement with previous morphological characterizations of *in vitro* neuronal networks at a single developmental stage [6], where a similar small-world arrangement of connections was evidenced at DIV 6. However, to reinforce the evidence of the emergence of a small-world configuration *during* the graph development (as well as the fact that here the small-world metrics are not influenced by network disconnectedness), we also measured the global and local efficiency, as introduced by Latora and Marchiori in [8]. These latter quantities, indeed, are seen as alternative markers of the small-world phenomenon, in that small-world networks are those propagating information efficiently both at a global and at a local scale. The efficiency curves of the cultured networks are reported in Fig. 4C as a function of age, and compared to the efficiency of the equivalent random graphs. Our results indicate that the connectivity structure of the neuronal networks evolve towards maximizing global efficiency (making it similar to the value of random graphs), while promoting fault tolerance by maximization of local efficiency (which is, instead, larger than the local efficiency of a random graph), and both properties are realized at a relatively low cost in terms of number of links (see again Fig. 3A).

### Node degree distribution evolution

**Figure 5:**
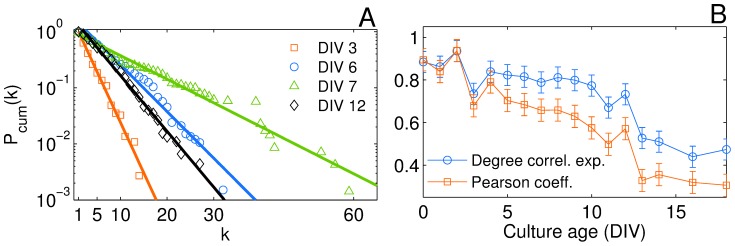
Degree distribution and degree-degree correlation. (A) Cumulative node degree distributions on a semi-log scale for the state of the same culture at DIVs 3, 6, 7, and 12 (see legend for the symbol coding). Solid lines correspond to the best exponential fitting 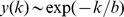, with 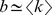 the mean degrees at DIV 3, 6, 7, and 12 respectively. (B) Degree correlation exponent (blue circles) measuring the network assortativity and the corresponding Pearson coefficient (red squares). Both quantities are averaged for the set of 6 cultures at each day of measure (DIV) and error bars represent the sem.

Turning now our attention to network statistics at the micro-scale, we investigated how the node degree distributions evolved during maturation process. At all ages, cultures appeared to belong to the class of single-scale networks, displaying a well defined characteristic mean node degree. Figure 5A shows that the cumulative degree distributions 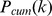 for DIVs 3, 6, 7, and 12 had a fast decay with a non monotonous increase in the average connectivity, with most of the nodes having a similar number of connections and only a few ones with degrees deviating significantly from such a number.

The data were fitted to an exponential scaling law 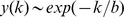 with a level of confidence larger than 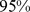. The values of the scaling parameter 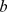 were close, within error, to the mean degree 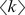 of the networks at each culture age. It has to be remarked that the distribution of node connections, although always homogeneous, shifted during culture maturation toward much broader distributions, with few highly connected nodes appearing at DIVs 6 and 7. These “hubs” at the peak days of the culture evolution result from a branching process, allowing each single neuron to reach a larger neighborhood. Thus, at variance with scale-free networks [22], [23], our cultured and clustered networks are identified as a single-scale homogeneous population of nodes. This is in agreement with reports on many other biological systems like the neuronal network of the worm *Caenorhabditis elegans* [24], [25], and suggests the existence of physical costs for the creation of new connections and/or nodes limited capacity [26].

While the number of neighbors (the degree) is a quantity retaining information at the level of a single node, one can go further to inspect degree-degree correlations, i.e. to quantify whether the degrees of two connected nodes are correlated. It is known, indeed, that biological networks feature generally disassortative network structures [27], that is connections are more likely to be established between high-degree and low-degree nodes. In our system, we used the assortativity coefficient described in the Experimental set-up section. Figure 5B shows the age evolution of the Pearson coefficient 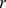 and of the exponent 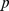 that characterizes the scaling behavior of the degree correlation properties (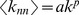). At one hand, as 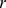 stays positive during the whole development we can generally conclude that our networks are assortative and, on the other hand, the trend of the exponent 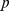 indicates that there is a transition from an almost linear (from DIV 0 to DIV 2) to a sub-linear (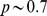) degree-degree correlation regime during the small-world stage.

It is important to remark here that, to the best of our knowledge, this is the first report of assortativity in an *in vitro* cultured neuronal network, and such an evidence actually links to other studies where assortativity was found in simple *in vivo* neuronal systems, like the *C. elegans* neural network structure [28].

### Spatial-growth model

A series of previous studies [15], [16] singled out tension along neurites and adhesion to the substrate as the two main factors conditioning the neuronal self-organization into a clustered network. Here we go a step ahead, and propose a simple spatial model which not only incorporates migration of neurons but also explicitly considers neurite growth, and the establishment of synaptic connections.

**Figure 6:**
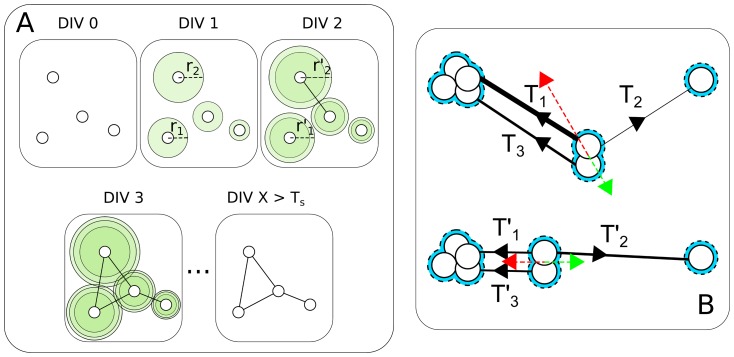
Growth model. (A) Schematic representation of how cells get connected. At DIV 0, 4 cells of radius 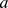 are located at random positions. The first iteration of the algorithm, DIV 1, assigns to each cell 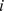 a disk of radius 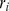 (green shade). At the next iteration, DIV 2, the disk's growth rate decreases, 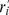, and a link between two cells is established when their disks intersect (DIV 3). This process continues until 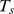 steps. (B) Force diagram explaining cell migration and clustering. Tension forces 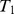, 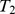, and 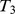 are acting on the central cluster composed of two cells, whose vector sum (red arrow) exceeds the adhesion to the substrate (green arrow). As a result, a new equilibrium state is produced with new tension forces 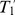, 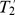, and 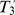, being the central cluster pulled in the direction of the net force approaching the largest cluster.

Our model is schematically illustrated in Fig. 6. We start by considering a set of 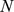 cells. Each cell is a small disk of radius 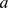 randomly distributed in a 2-dimensional circular substrate of area 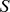. The algorithm then evolves the connections and positions of such disks at discrete times, each time step 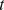 corresponding to a DIV of the culture. The complex process of neurite growth and the establishment of synaptic connections is modeled by associating to each cell a time growing disk in such a way that, two cells are linked at a given time step if their outer rings intersect as shown for DIV's 2 and 3 in Fig. 6A. At each time step 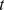, the radius 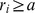 increases by a quantity 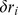 which decays as
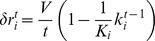
where 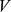 is the neurite growth velocity (the same for all cells), 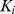 a random number in the interval 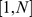 and 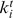 the degree of the node (cell) at the time step 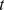. The term 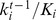 introduces heterogeneity in the cell population, and represents the fraction of links acquired by the cell in the previous steps from the initial randomly assigned endowment 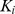. A very large 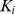 indicates that, potentially, a cell is very active and could connect many other cells. The wiring process is iterated up to a given time step 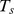, at which the formation of new connections is stopped.

As for the process of cell migration and clusterization, cells or clusters whose distance is less than 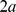 are then merged into the same new cluster. Furthermore, whenever two cells are connected, an initial tension 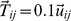 is created between them, and it is incremented in 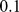 force units at each time step, being 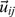 the unit vector along the direction connecting the two cells. The total force acting on a cell or cluster 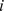 is given by 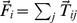 with 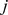 running over the cell indexes connected to 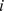, and not belonging to the same cluster. Furthermore, each cell is “anchored” to the substrate by a force 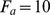 force units, and the i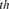 cell can only be detached if there is a net force 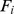 acting on it larger than 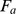. In the case of a cluster of cells, the adhesion force to the substrate is considered to be the sum of the individual adhesions of the cells composing the cluster. Therefore, cells and clusters move in a certain direction in all circumstances in which the net force acting on them overcomes the adhesion force, and an equilibrium point is reached at a new position in which the new net force balances (or is smaller) than the adhesion to the substrate (see Fig. 6B).

In order to validate our model, we ran a large number of simulations for different values of the model parameters 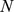, 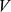 and 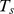. Remarkably, when comparing the statistical topological features of the simulated networks to those measured from the set of 6 cultures, we found high correlation values exist only in a very narrow window of 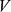 and 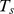. For instance, the parameter values which better fit the experimental observations for 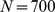 are 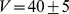 and 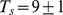.

**Figure 7:**
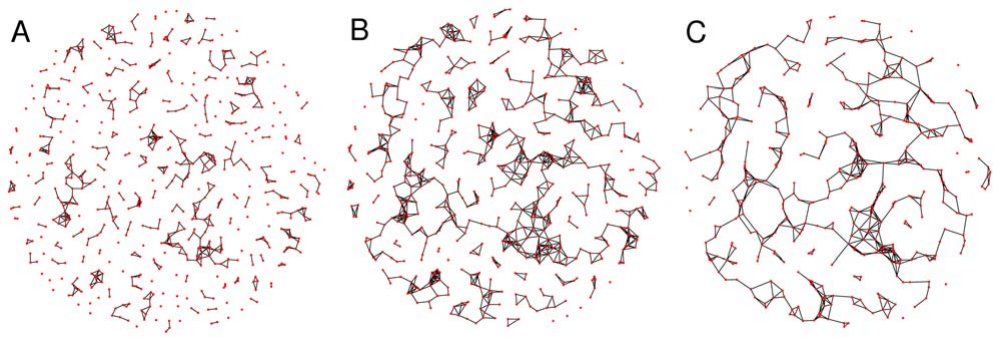
Schematic illustration of the network self-organization at three different instants of the automata generations. (A) DIV 3, (B) DIV 6, and (C) DIV 12. Simulation parameters: 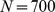, 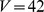, and 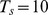.

Figure 7 shows a typical output of the evolution of a simulated network. The initial number of cells is taken to be of the same order as in the experiments, and we chose as parameters 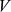 and 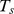 those with the highest correlation with experiments. Boundary conditions mimic the real experimental setup by canceling the adhesion force to the substrate outside the culture area. The spatial organization of the network of cells and clusters after 3, 6, and 12 DIV, closely resembles the one observed in the experiments (see Fig. 1).

**Figure 8:**
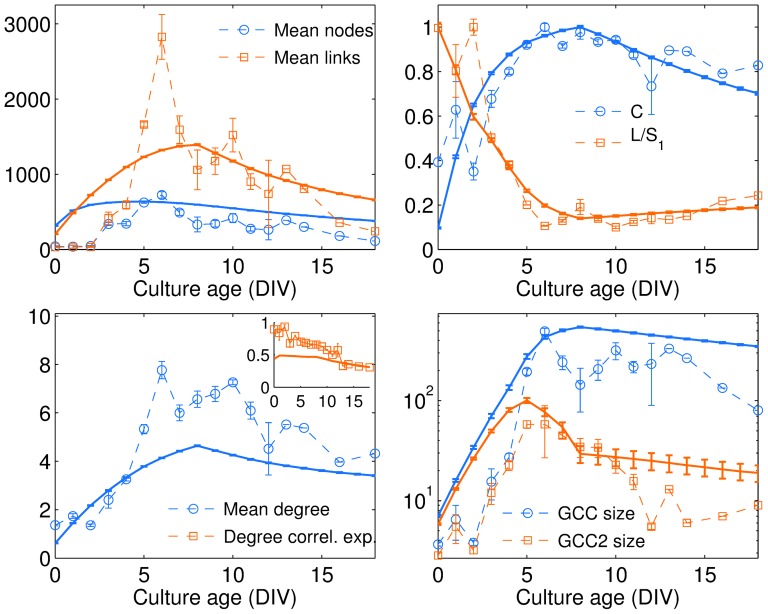
Comparison between model and experiment. Legends in each panel clarifies on the topological quantities measured in experiments (dashed curves), and the corresponding trends of the simulated networks (solid curves). Simulation parameters are the same as in the caption of Fig. 7, and each point is the ensemble average over 50 independent runs of the growth algorithm.

Despite its relative simplicity, it is remarkable that the model offers a rather good qualitative verification of the trends of all the structural network characteristics measured in the experiments. In particular, Fig. 8 reports a synoptic comparison of the mean number of nodes and links, of 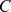 and 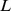, of the mean degree and degree correlation, and of the sizes of the GCC and GCC2, measured in the experiments and those obtained from the simulations of the model with 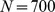, 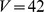, and 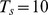. The main observed difference is found in the mean degree, the reason behind such a slight discrepancy being that the model does not include any neurite branching process, limiting the size of the neighborhood encompassed by the nodes.

Though it would have been unrealistic to expect a perfect quantitative agreement between model and observations, the fact that the model reproduces the main qualitative scenarios of the experiments indicates that it captures the main processes underlying the observed morphological evolution and self-organization of the cultures.

## Discussion

In summary, we provided a large scale experimental investigation of the morphological evolution of *in vitro* primary cultures of dissociated invertebrate neurons from locust ganglia. At all stages of the culture's development, we were able to identify neurons' and neurites' location in automated way, and extract the adjacency matrix that fully characterizes the connectivity structure of the networking neurons. A systematic statistical analysis of a group of topological observables has later allowed tracking of the main network characteristics during the self-organization process of the culture, and drawing important conclusions on the nature of the processes involved in the culture' structuring. At early stages of development (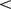
DIV 3) characterized by a high neurite growth rate, homogeneous node degree distribution and low clustering resulted in a random topology as expected given the fact that neurons were randomly seeded. Following this immature period, neurite growth rate diminished and tension along neurites started to shift the network to a small-world one with path lengths similar to random configurations but presenting high clustering of connections. This transition from random to small-world concurred with the percolation of the culture and the onset of the giant connected network component.

Furthermore, the identification of the main physical processes taking place during the culture's morphological transformations, allowed us to embed them into a simple growth model, qualitatively reproducing the overall scenario observed in the experiments.

Our results extend previous studies where network properties of cultures were investigated at a particular developmental stage and for a lesser number of nodes [6]. These results also systematically characterize several topological network measures along the entire culture's evolution, and unveil many yet unknown self-organization properties, such as *i*) the fact that a small-world configuration spontaneously emerges in connection to a large increase in both local and global network's efficiency, and *ii*) the evidence that cultures tend to organize in a regime of non trivial degree mixing which, in turn, is characterized by assortative degree-degree correlation features. The evolution from an initial random to a small-world topology has also been reported recently in the context of a functional network of a cortical culture [13]. However, although functional connectivity correlates well with anatomical connectivity, there are studies showing that strong functional connections may exist between nodes with no direct physical connection [12]. This suggests that future studies are needed in which both anatomical and functional networks are accessible in order to understand their complex entanglement.

Given the absence of external chemical or electrical stimulations, we conclude that such complex network evolution and morphological structuring is indeed an intrinsic property of neuronal maturation. Our study therefore contributes to the understanding of the complex processes ruling the morphological structuring of cultured neuronal networks as they self-organize from collections of separated cells into clustered graphs, and may help identifying culture development stages in new, specific and targeted, experiments.
